# Sight restoration reverses blindness-induced cross-modal functional connectivity changes between the visual and somatosensory cortex at rest

**DOI:** 10.3389/fnins.2022.902866

**Published:** 2022-09-23

**Authors:** Negin Nadvar, Noelle Stiles, Jeiran Choupan, Vivek Patel, Hossein Ameri, Yonggang Shi, Zhongming Liu, John Jonides, James Weiland

**Affiliations:** ^1^Department of Biomedical Engineering, University of Michigan, Ann Arbor, MI, United States; ^2^Laboratory of Neuro Imaging, USC Mark and Mary Stevens Neuroimaging and Informatics Institute, Keck School of Medicine of USC, University of Southern California, Los Angeles, CA, United States; ^3^Irvine School of Medicine, The University of California, Irvine, Irvine, CA, United States; ^4^Department of Electrical Engineering and Computer Science, University of Michigan, Ann Arbor, MI, United States; ^5^Department of Psychology, University of Michigan, Ann Arbor, MI, United States; ^6^Department of Ophthalmology and Visual Sciences, University of Michigan, Ann Arbor, MI, United States

**Keywords:** resting-state functional connectivity, blindness, sight restoration, cross-modal plasticity, retinal prosthesis, fMRI

## Abstract

Resting-state functional connectivity (rsFC) has been used to assess the effect of vision loss on brain plasticity. With the emergence of vision restoration therapies, rsFC analysis provides a means to assess the functional changes following sight restoration. Our study demonstrates a partial reversal of blindness-induced rsFC changes in Argus II retinal prosthesis patients compared to those with severe retinitis pigmentosa (RP). For 10 healthy control (HC), 10 RP, and 7 Argus II subjects, four runs of resting-state functional magnetic resonance imaging (fMRI) per subject were included in our study. rsFC maps were created with the primary visual cortex (V1) as the seed. The rsFC group contrast maps for RP > HC, Argus II > RP, and Argus II > HC revealed regions in the post-central gyrus (PostCG) with significant reduction, significant enhancement, and no significant changes in rsFC to V1 for the three contrasts, respectively. These findings were also confirmed by the respective V1-PostCG ROI-ROI analyses between test groups. Finally, the extent of significant rsFC to V1 in the PostCG region was 5,961 in HC, 0 in RP, and 842 mm^3^ in Argus II groups. Our results showed a reduction of visual-somatosensory rsFC following blindness, consistent with previous findings. This connectivity was enhanced following sight recovery with Argus II, representing a reversal of changes in cross-modal functional plasticity as manifested during rest, despite the rudimentary vision obtained by Argus II patients. Future investigation with a larger number of test subjects into this rare condition can further unveil the profound ability of our brain to reorganize in response to vision restoration.

## Introduction

Visual impairment has a negative effect on the quality of life of those afflicted by limiting their day-to-day activities, including environmental engagement and social opportunities. Vision rehabilitation teaches skills that help them play a more active and satisfying role in society (Bourne et al., 2012; Khorrami-Nejad et al., 2016; National Academies of Sciences Engineering and Medicine et al., 2016); however, rehabilitation does not improve vision itself. Sight restoration approaches have reached clinical trials and, in some cases, regulatory approval. Gene therapy (Bennett et al., 2016; Ashtari et al., 2017; Apte, 2018; Lee et al., 2019; Wang et al., 2020), optogenetic (McClements et al., 2020; Simon et al., 2020), and retinal prostheses (Zrenner et al., 2011; Fernandes et al., 2012; Stingl et al., 2013; Zrenner, 2013; Cheng et al., 2017; BLoch et al., 2019) have all been tested in patients with retinal disease. The degree of vision restoration provides patients with improved mobility and object detection. However, in most cases, recipients of these new therapies still have a significant visual impairment despite the regained function. While behavioral experiments that assess functional vision in real-world scenarios are the most important endpoints, measures of the cortical response to vision restoration can provide complementary information that explains clinical outcomes and guides future development of improvements in these therapies.

A need for biomarkers that help gauge patients’ improvement during post-sight restoration rehabilitation becomes consequential. The functional magnetic resonance imaging (fMRI) blood-oxygen-level dependent (BOLD) activation response has been utilized for assessing brain plasticity following sight restoration. One study showed that prolonged use of the Argus II implant increased the fMRI BOLD response to visual stimuli in the primary visual cortex (Castaldi et al., 2016). Another neuroimaging experiment performed on retinal gene therapy patients demonstrated significantly enhanced fMRI activation in the visual cortex in response to visual checkerboard stimulation (Ashtari et al., 2017) compared to the same measurement made in the same individuals before therapy.

Resting-state functional connectivity (rsFC) has been used to gauge the plastic changes in the brain following sensory deprivation. This approach has been extensively studied with different analysis methods (Biswal et al., 1995; Bullmore and Sporns, 2009; Li et al., 2009; van den Heuvel and Hulshoff Pol, 2010; Van Dijk et al., 2010; Peltier and Shah, 2011; Barkhof et al., 2014; Iraji et al., 2016; Lv et al., 2018; Seitzman et al., 2019). The rsFC analysis reveals the relationship between spontaneous brain activity in different parts of the brain in the absence of any cognitive or sensory stimulation. Numerous rsFC studies have examined alterations in functional connectivity between the visual cortex and other brain sensory or cognitive areas following vision loss. This literature robustly shows that following visual sensory deprivation in congenital, early, and late blindness, rsFC decreases both within the visual cortical areas (Liu et al., 2008; Dai et al., 2013; Qin et al., 2014; Heine et al., 2015; Murphy et al., 2016; Hou et al., 2017; Huang et al., 2019; Hu et al., 2020) and between the visual cortex and other sensory (somatosensory or auditory) cortices (Wittenberg et al., 2004; Liu et al., 2008; Yu et al., 2008; Dai et al., 2013; Striem-Amit et al., 2015; Murphy et al., 2016; Bauer et al., 2017; Wen et al., 2018; Huang et al., 2019). In contrast, the rsFC between the visual cortex and cognitive regions of the brain has been shown to have enhanced the following vision loss (Heine et al., 2015; Striem-Amit et al., 2015; Murphy et al., 2016; Sabbah et al., 2016, 2017; Bauer et al., 2017; Wen et al., 2018; Hu et al., 2020); a phenomenon that has been attributed to increased top-down influence in the visual cortex due to visual deprivation.

In contrast to the abundant literature supporting the functional plastic changes in the brain following blindness, few studies have examined alterations in functional connectivity after sight restoration. A single analysis study on the effect of sight recovery 3 years after gene therapy application showed that rsFC between the visual and auditory areas was enhanced after sight restoration, partially reversing the effect of blindness (Mowad et al., 2020) and supporting the feasibility of using rsFC as a biomarker of vision restoration. To further investigate the relationship between rsFC and vision restoration, we studied functional connectivity in a cohort of patients with retinitis pigmentosa (RP) who were implanted with the Argus II retinal prosthesis to determine if this treatment, which partially restores vision, also reverses, in full or in part, the plastic changes induced by the vision loss.

## Materials and methods

### Human subjects

A total of 27 subjects were included in the analysis and divided into three groups: 10 healthy controls (HC-5 women, age 54.50 ± 13.84), 10 RP blind (RP-3 women, age 51.10 ± 12.92), and 7 Argus II subjects (3 women, age 64 ± 9.71)—the difference in age among the groups was insignificant (F2, 24) = 2.40, *p* = 0.11). Details of subjects’ demographic and clinical information are included in Supplementary Tables 1, 2. The RP subjects were all blind with visual acuity of worse than 20/200 and a visual field of less than 20°, except for subject 13, which had a visual acuity of 20/80-2 and 20/50 + 2 and a visual field of 2° or less in both eyes. The Argus II subjects were legally blind from RP. Their baseline vision was bare light or no light perception, per the FDA-approved indication for Argus II. Out of the 27 subjects, 20 were recruited and consented to at 2 Human Connectome for Low Vision (HCLV) data collection centers per the approved Institutional Review Board at each center: the University of Michigan (UM) and the University of Southern California (USC); this included 3 HC, 10 RP, and 5 Argus II subjects at USC and 2 Argus II subjects at UM. The remaining 7 HC subjects’ data was sourced from the Human Connectome Project for Aging (HCP-A) public database (Bookheimer et al., 2019). One additional Argus II subject’s neuroimaging data were collected at USC. However, the subject was determined to be a significant outlier and was removed from the analysis. For more details, please see the Subject Outlier Identification section in Supplementary Material. Resting-state functional runs showing the subject motion of more than 1 mm in any of the x, y, or z directions were excluded from the analysis. Moreover, the resting-state runs were included in the analysis only if they were acquired at the beginning of the sessions before any other task performance. These criteria excluded 22 out of 112 runs from the analysis among all the subjects in all the sessions.

### Argus II retinal prosthesis

The Argus II retinal prosthesis is an epiretinal implant that was the first retinal prosthesis with FDA approval obtained in 2013 and CE approval in 2011. The system comprises an internal implant unit and an externally worn unit. The internal system contains an intraocular array with an area of 3.5 by 6 mm, covering an area of 11 × 19 degrees of the visual field. The array is a 6 × 10 grid of platinum surface electrodes that are 200 μm in diameter and spaced 575 μm apart. The electronic supporting case is sutured to the sclera and inductively receives power/data from the external system. The external unit contains a video camera mounted on a pair of glasses worn by the patient, a video processing unit (VPU), and a battery. The camera transmits the visual data to the VPU, where data are processed, sent to the external coil, and relayed to the internal circuitry. RP patients using Argus II could perceive motion (Dorn et al., 2013) and showed the best visual acuity of 20/1,260 (Humayun et al., 2012).

### Experimental paradigm

The UM, USC, and HCP-A data neuroimaging experiments followed the same paradigm. Each scan visit was composed of two scanning sessions separated by a break. The MRI scans comprised structural and resting-state fMRI (rsfMRI) scans. During the rsfMRI runs, subjects were asked not to engage in any tasks while lying in the scanner. Upon completing the T1W scan, the technicians reviewed the quality of the structural images captured during the first scan session. A re-scan was performed during the second scanning session if the scan quality was deemed low from the first session. The participants completed a total of four runs of rsfMRI with their eyes open under a dark foam mask at UM and USC. This was performed due to the inability of blind subjects to fixate. For HCP-A rsfMRI runs, healthy controls were asked to fixate while lying in the scanner.

### Data acquisition

Each subject acquired one anatomical and four resting-state functional runs (2 runs per scan session). Structural MRI scans at USC were acquired using a 3T Siemens Prisma scanner. The T1W structural scans used MPRAGE (Magnetization Prepared Rapid Gradient Echo) 3D acquisition, voxel dimension 0.8 × 0.8 × 0.8 mm^3^, TI (inversion time)/TE (echo time)/FA (flip angle) = 1,000 ms/2.22 ms/8°. The resting-state functional runs were obtained with 2D gradient-echo (GRE) echo-planar imaging (EPI) acquisition with multiband (MB) acceleration factor = 8, voxel size of 2 × 2 × 2 mm^3^ with TR (repetition time)/TE/FA = 800 ms/37 ms/52° with a total of 420 volumes for each run. At UM, T1W images were obtained with a 3T GE MR750 scanner with 3D spoiled gradient echo (SPGR) with inversion recovery magnetization preparation, voxel size of 0.5 × 0.5 × 0.8 mm^3^, TI/TE/FA = 1,060 ms/Min Full/8° (“Min Full” refers to the minimum TE to obtain full echo acquisition). Functional runs were acquired with interleaved GRE-EPI, MB = 6, voxel size 2.4 × 2.4 × 2.4 mm^3^ and TR/TE/FA = 800 ms/30 ms/52°. Field maps were acquired and used to correct the geometric distortion in EPI due to magnetic field inhomogeneities. Scan parameters for HCP-A structural and functional data were identical to USC parameters (Harms et al., 2018). Harmonization of the data between the HCLV collection centers at USC and UM was investigated using the data from a traveling subject and a Function Biomedical Informatics Research Network (fBIRN) phantom as a part of a previous study (Nadvar et al., 2019). Refer to Supplementary Material for a summary of the results of this analysis.

### Data preprocessing

The field inhomogeneity inside the scanner (field map) was calculated with the FSL Topup function and an in-house Linux bash and MATLAB script. Using the Realign and Unwarp functions in combination with the field map toolbox in SPM, the susceptibility distortion, motion artifact, and susceptibility-by-movement interaction were corrected. The first 12 volumes were removed to ensure reaching a steady state (and additionally, the last 46 volumes were removed for HCP-A data due to a different length), yielding 420 volumes for each functional run. The reference volume for motion and field map correction was the 10th for UM data and the single-band reference volume (SBRef) for USC and HCP-A data. Motion correction parameters were created and used as regression covariates. The origin of the structural and functional images was manually set at the anterior commissure to enhance the outcome of the co-registration and normalization to the standard template. Potential outlier volumes were flagged using the MATLAB CONN toolbox (Whitfield-Gabrieli and Nieto-Castanon, 2012) with the global BOLD signal above 5 standard deviations or frame-wise displacement of more than 0.9 mm, creating the scrubbing regression covariate. Using indirect segmentation and normalization in CONN, the functional and anatomical images were first co-registered using an affine transformation. The structural image was then normalized to standard MNI space and segmented into gray matter (GM), white matter (WM), and cerebrospinal fluid (CSF). This process continued iteratively until convergence, yielding non-linear spatial transformation parameters that were then applied to both anatomical and functional images; these images were resampled to isotropic 1 mm and 2 mm voxels, respectively. Functional images were spatially smoothed with a Gaussian kernel with a 4 mm full-width-at-half-maximum (FWHM). The anatomical component-based noise correction (aCompCor) was also implemented using CONN to minimize physiological noise further; the first five principal components of the BOLD in WM and CSF were extracted to be used as confounding effects. All the confounding factors, including the six translation/rotation motion parameters and their derivatives, scrubbing covariates, and WM/CSF covariates, were linearly regressed out of the BOLD signal. Finally, the time series were bandpass filtered between 0.008 and 0.0 Hz and linearly detrended.

### Statistical analysis

Seed-based connectivity (SBC) maps were calculated using CONN; bivariate Pearson’s correlation coefficients between the average BOLD signal in primary visual cortex (V1) Region of Interest (ROI) and the BOLD time series in the rest of the brain were first computed and Fisher z-transformed. Group-level SBC maps were created by applying a one-sample *t*-test to Fisher’s *z* values associated with each voxel across subjects. A two-sample *t*-test was used for contrast analysis between the groups. The second-level analysis results were corrected for multiple comparisons using cluster-level inference to control for false positives. Parametric statistics were applied using Gaussian Random Field theory (Worsley, 1996) with an uncorrected voxel threshold of *p* < 0.001 and a cluster-level threshold of *p* < 0.05 for cluster size false discovery rate (FDR) correction. The ROI-ROI functional connectivity between V1 and post-central gyrus (PostCG) was also calculated as Fisher-transformed correlation coefficients between these 2 ROIs in BOLD time series, representing the effect size. Additionally, we looked at the spread of significant FC in a target ROI. This was defined as the cortical volume (in mm^3^) with a statistically significant connection to the seed in group-level FC maps corrected at the cluster level. Considerations of data normality, effect size, and power are further investigated as a part of section 5 in Supplementary Material.

### ROI selection

The 2020 Julich-Brain atlas (V2.9), an intricate volumetric atlas based on the cytoarchitecture of the brain (Amunts et al., 2020), was used to define ROIs. Given our *a priori* hypothesis regarding the primary visual and somatosensory cortex, we extracted these 2 ROIs from the atlas: the V1 was formed by combining the respective left and right ROIs from the atlas. PostCG, the location of the primary somatosensory cortex, was defined by merging the areas 1, 2, 3a, and 3b on the left and right sides as defined by the atlas.

## Results

### How does retinitis pigmentosa blindness affect functional connectivity to V1 at rest?

**FIGURE 1:**
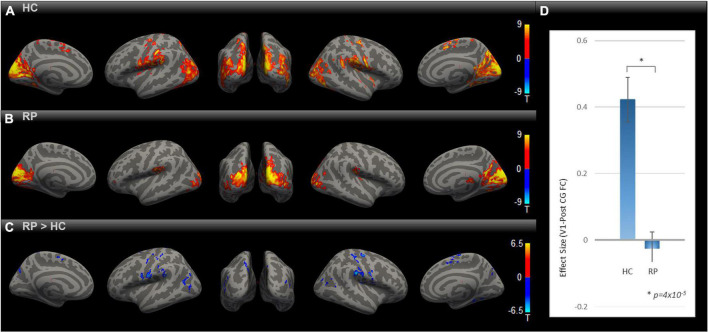
Comparing rsFC between RP and HC. Group-level rsFC maps using V1 as the seed for HC **(A)**, RP **(B),** and RP > HC contrast **(C)** were corrected for multiple comparisons using Gaussian Random Field Theory with an uncorrected voxel-level threshold of *p* < 0.001 and a cluster-level threshold of *p* < 0.05 FDR corrected for cluster size. Areas with lower rsFC to V1 covered regions of higher-level visual, primary/secondary somatosensory, motor, and parietal association cortex [blue blobs in **(C)**]. V1-to-PostCG ROI analysis **(D)** showed significantly lower rsFC effect size in RP vs. HC, using two-sample *t*-test [*t* (18) = –5.39, *p = 4 × 10^–5^].

In order to evaluate the impact of blindness on functional connectivity with the primary visual cortex, we obtained the SBC maps for HC and RP groups as well as the between-group contrast as indicated in Figures 1A–C), each corrected for multiple comparisons using Gaussian Random Field Theory with an uncorrected voxel-level threshold of *p* < 0.001 and a cluster-level threshold of *p* < 0.05 FDR corrected for cluster size. In both the HC and RP groups, V1 showed strong functional connectivity to different parts of the occipital cortex, primary and secondary somatosensory cortex, and inferior temporal areas. Blue color-coded regions in Figure 1C highlight areas in the RP group with significantly lower functional connectivity to V1 than HC. In the indicated seed-to-voxel functional connectivity map in Figure 1C, these areas overlap with parts of higher-level visual areas such as the cuneus and lateral occipital cortex, as well as regions in primary and secondary somatosensory areas, motor cortex, and some parietal association areas. In the ROI-to-ROI analysis (Figure 1D) between V1 and PostCG, the effect size (Fisher z-transformed correlation coefficient value) was significantly lower in RP compared with HC [*t* (18) = –5.39, *p* = 4 × 10^–5^].

### How does partial sight restoration with Argus II alter functional connectivity to V1 at rest?

**FIGURE 2:**
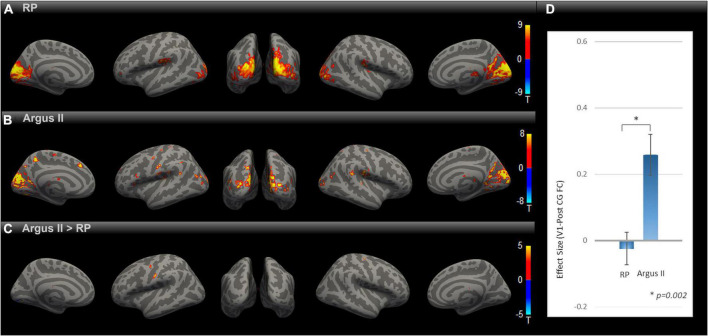
Comparing rsFC between Argus and RP. Whole-brain rsFC map using V1 as the seed is calculated at the group level for RP **(A)** Argus II **(B)** and contrast Argus II > RP **(C)**. rsFC maps were corrected for multiple comparisons using the Gaussian Random Field Theory with an uncorrected voxel-level threshold of *p* < 0.001 and a cluster-level threshold of *p* < 0.05 FDR corrected for cluster size. Areas with higher rsFC in Argus II than RP involved pre- and PostCG regions. The ROI-to-ROI rsFC analysis between V1 and PostCG **(D)** additionally showed significant [*t* (15) = 3.62, *p = 0.002] enhancement of this connectivity in Argus II compared with RP.

We evaluated how partial sight restoration with Argus II can alter intrinsic brain connectivity in RP blindness. SBC maps for RP and Argus II (Figures 2A,B) groups were calculated and corrected for multiple comparisons with an uncorrected voxel-level threshold of *p* < 0.001 and a cluster-level threshold of *p* < 0.05 FDR corrected for cluster size. Both groups demonstrated functional connectivity between V1 and other visual and inferior temporal areas. The contrast map between the two groups, corrected for multiple comparisons, is shown in Figure 2C. Intriguingly, the seed-to-voxel functional connectivity map in Figure 2C revealed parts of primary motor and somatosensory (pre- and PostCG) with enhanced rsFC to V1 in the Argus II compared with the RP group. The ROI-to-ROI analysis between our two areas of interest (V1 and PostCG) in Figure 2D showed that the functional connectivity significantly increased after partial sight restoration with Argus II [*t* (15) = 3.62, *p* = 0.002].

### How close are the results in the sight-restored to normally sighted?

**FIGURE 3:**
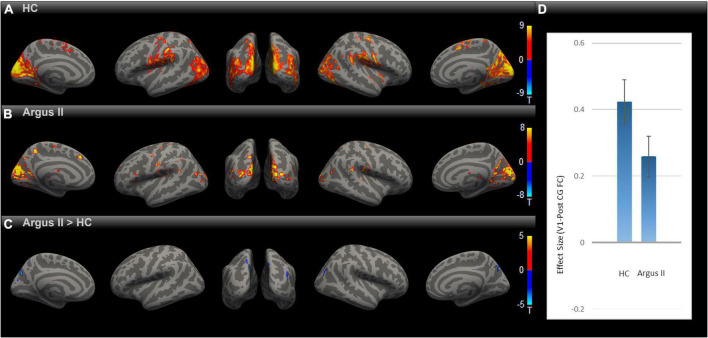
Comparing rsFC between Argus II and HC. Using the V1 seed, the group-level rsFC maps for Argus II **(A)**, HC **(B),** and Argus II > HC contrast **(C)** were corrected for multiple comparisons using the Gaussian Random Field Theory with an uncorrected voxel-level threshold of *p* < 0.001 and a cluster-level threshold of *p* < 0.05 FDR corrected for cluster size. The contrast map shows that the areas depicting lower rsFC to V1 were found in some higher-level visual areas (shown in blue). The ROI-to-ROI rsFC analysis **(D)** revealed no significant difference in the V1-PostCG rsFC effect size between Argus II and HC groups, *t* (15) = –1.72, *p* = 0.1.

Having observed enhanced rsFC after sight restoration, as demonstrated in Figure 2, an intriguing question is whether this was a partial or full reversal compared to normally sighted individuals. To that end, we compared the functional connectivity maps for HC and Argus II groups (Figures 3A–C) after correcting for multiple comparisons using Gaussian Random Field Theory with an uncorrected voxel-level threshold of *p* < 0.001 and a cluster-level threshold of *p* < 0.05 FDR corrected for cluster size. The seed-to-voxel functional connectivity map in Figure 3C shows that sight restoration with Argus II was not able to fully reverse the blindness-induced decrease in rsFC within the visual cortex, especially between V1 and higher visual areas, as indicated by small blue color-coded regions in the medial and lateral sides of the higher-level occipital cortex. Interestingly, partial sight restoration reversed alterations in V1 functional connections with the pre-and PostCG. As indicated in Figure 3D, ROI-to-ROI functional connectivity between V1 and PostCG showed no significant difference between the HC and Argus II groups in a two-sample *t*-test with *t* (15) = –1.72, *p* = 0.10.

### Effect of blindness and sight restoration on the spread of significant functional connectivity to V1

**FIGURE 4:**
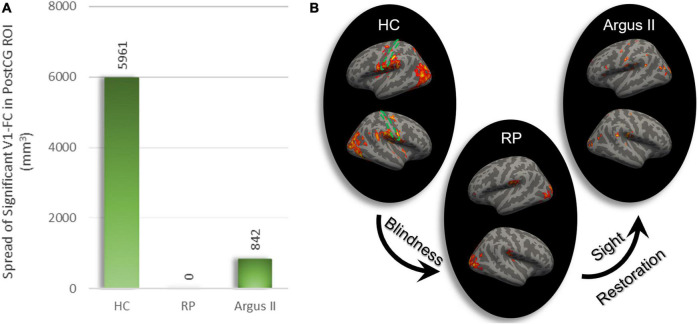
The extent of rsFC to V1. The volumetric extent in PostCG shows significant rsFC to V1 as the seed was calculated in mm^3^ **(A)**. This spread of connectivity drastically reduced after blindness and partially increased following sight restoration in Argus II. Areas in the whole brain with significant rsFC to V1 for each of the three study groups are shown on the right **(B)**. The dotted green area represents PostCG/primary somatosensory cortex boundary.

In order to evaluate how blindness and sight restoration affect functional connectivity, one approach is to look at the strength of this connection, as described in Figures 1–3. Another way of evaluating such alterations is to consider how broadly the connectivity patterns spread over a target ROI. To examine this, we defined another metric that measures the extent of significant functional connectivity as the volumetric area in the target ROI with a significant functional connection to the source ROI. Using V1 as the source ROI and PostCG as the target ROI, we computed the extent of significant connections for HC, RP, and Argus II groups, as indicated in Figure 4B. Figure 4A shows that the calculated volumetric spread of functional connectivity to V1 in PostCG was 5,961 mm^3^ in the HC group. This metric decreased to 0 mm^3^ for RP and then increased to 842 mm^3^ for the Argus II group.

## Discussion

This study investigated how the loss and subsequent re-introducing of visual input can affect visual-somatosensory functional connectivity at rest. At the group level, the healthy controls demonstrated somatosensory cortex areas with significant rsFC to V1. In the blind RP group, these regions were significantly reduced in rsFC with V1. Additionally, we evaluated the effect of partial sight restoration with the Argus II retinal prosthesis at the group level. The results clearly indicated a change in the opposite direction as a significant increase in visual-PostCG rsFC in Argus II compared with the blind RP group. Importantly, this level of increase rendered the somatosensory-visual rsFC in the Argus II group at a level close to the healthy controls, as no significant rsFC was observed between the two regions in the contrast analyses, with either connectivity maps or ROI analyses. Additionally, the extent of regions in the somatosensory cortex with significant rsFC to V1 followed the same direction as the strength: reduction in the RP blind compared with HC and enhancement in Argus II vs. the fully blind RP group.

We focused on visual-somatosensory rsFC. This choice allowed us to take advantage of the many prior studies of the effects of blindness on visual-somatosensory cross-modal plasticity, against which we could compare our findings. We observed that RP blindness reduces visual-somatosensory rsFC; this finding is consistent with many prior experiments that evaluated rsFC in late-blind individuals (Wittenberg et al., 2004; Dai et al., 2013; Murphy et al., 2016; Wen et al., 2018; Huang et al., 2019). There is also similar evidence for this reduction in rsFC in the congenitally (Striem-Amit et al., 2015; Murphy et al., 2016) and early blind (Wittenberg et al., 2004; Liu et al., 2008; Bauer et al., 2017). Strikingly, some other studies on the blind observed the involvement of the visual cortex in the processing of language and mathematics as well—examples of higher-level cognitive tasks (Bedny et al., 2011; Kanjlia et al., 2016). rsFC has been shown to increase between visual and cognitive areas following blindness (Heine et al., 2015; Striem-Amit et al., 2015; Murphy et al., 2016; Sabbah et al., 2016, 2017; Bauer et al., 2017; Wen et al., 2018). Such an effect on functional connectivity following vision loss has been attributed to an increase in the top-down impact on the visual cortex. One model proposed to explain these unexpected alterations is the reverse hierarchy. In normally sighted individuals, as information is fed through the visual hierarchy, more and more complex and abstract visual features will be processed, which will then serve as an input to cognitive regions such as attention and decision. The feedback connection from higher to lower regions in the visual hierarchy serves to enforce selective attention and learning. It has been proposed that in early-blind individuals, this reverse connection serves to further elaborate information from higher-level visual areas and provide an input containing abstract information into the lower-level visual areas, giving rise to cognitive processing in these regions. Reverse hierarchy, however, has its own limitations in explaining the observed cognitive processing in the visual cortex (Bareket et al., 2017; Fine and Park, 2018).

Previous studies have attempted to evaluate brain alteration following visual restoration using fMRI BOLD activation as the metric. A study looked at the tactile-evoked cross-modal BOLD responses in occipital regions in two Argus II prosthesis patients (Cunningham et al., 2015), one at 5 weeks and the other at 15 weeks following implantation. The qualitative assessment of their results indicated that the strength and extent of activation in these subjects seemed to be affected by the time since implantation in these two cases, with the subject with a longer time post-surgery demonstrating tactile-evoked visual activation levels closer to the group with normal vision. However, given that only two subjects were evaluated, no firm conclusions can be drawn from this study. Another study evaluated auditory-evoked cross-modal BOLD responses in patients whose sight was restored using RPE65 gene therapy technology (Mowad et al., 2020). Their qualitative comparison of the study groups discovered that the baseline blind RPE65 subjects had enhanced activations within the bilateral visual cortices due to auditory stimulation. However, 3 years later, these activation patterns were significantly elevated. Furthermore, in their study, the visual-auditory rsFC was qualitatively shown to be reduced in the baseline blind RPE65 subjects compared to healthy controls, whereas it slightly increased 3 years following gene therapy compared to baseline RPE65 subjects. Although the results were based on a qualitative comparison of activation maps between groups and not based on group-level contrast maps, their findings have important scientific implications.

Similar to gene therapy observations, our study revealed enhanced rsFC between V1 and PostCG following partial sight recovery with the Argus II retinal prosthesis. This represents a reversal of the cross-modal plasticity initially induced by blindness, as manifested at rest by using functional connectivity as the metric. The noted alteration in rsFC is remarkable in that it was observed even though retinal prosthesis patients regain only basic vision and only occasionally use their prosthetic implant. It is important to note that functional connectivity during rest was used as the metric in our study to investigate plastic changes in the brain following vision loss and restoration. How functional plastic changes manifest during task execution has been shown to be different from rest in blindness. In a study on early blind individuals, Pelland et al. (2017) looked at the apparent disagreement between task-dependent activation or connectivity and resting-state functional connectivity in the blind, i.e., an increase in cross-modal responses in the visual cortex during a non-visual sensory task and the decrease in rsFC between non-visual sensory and visual cortex. They hypothesized that such a decrease in rsFC in early blind individuals might be due to the involvement of the visual cortex in a larger number of processing modes during rest, when the brain is more available to explore various modes, resulting in an increase in functional connectivity variability at rest. On the other hand, increased functional connectivity during task execution might be due to brain involvement in limited modes, resulting in lower functional connectivity variability during the task. Therefore, it is essential to note that the rsFC for groups presented in our study does not smoothly generalize to the brain state during the execution of sensory tasks, such as tactile tasks. However, our finding shows that rsFC could be potentially used as a measure of functional plastic changes detectable during the resting state in response to visual sensory loss and restoration.

Our findings are limited by the relatively small number of participants. As such, we could not conduct a deeper analysis that might have revealed links between patient characteristics and rsFC. Recruitment is a challenge for any study, and Argus II patients are rare. Yet, a larger study that allows stratification and correlation analysis (using patient characteristics) has the potential to provide valuable information that can benefit the ongoing development of visual prostheses and other sight-restoration therapies. Factors such as device usage, duration of blindness, age, and time since implantation could all conceivably play a role in the brain’s functional organization after the therapeutic intervention. Our findings can serve as a starting point for such a study or be included in a meta-analysis of other similar studies that can increase the robustness of the results through a pooling of data.

## Conclusion

We showed that decreases in resting-state functional connectivity due to blindness were partially reversed by vision restoration. Despite advancements in vision restoration technologies, the vision provided remains well below healthy vision, and patient outcomes vary greatly. To better understand this variability, metrics associated with vision improvement are essential. The rsFC is a tool that has been broadly used to track functional changes in the brain following blindness. Studies of rsFC to investigate sight recovery are relatively rare. Our study aimed to evaluate the effect of blindness on visual-somatosensory rsFC and to further track changes in this quantity after partial sight restoration with the Argus II retinal prosthesis. Our investigation showed that visual-somatosensory rsFC has the potential to serve as a biomarker for functional plastic changes in the brain following vision recovery.

## Data availability statement

The datasets presented in this study can be found in online repositories. The names of the repository/repositories and accession number(s) can be found below: The Retinitis Pigmentosa (RP) and Argus II Human Connectome for Low Vision (HCLV) data that support the findings of this study have been provided to the Connectome Coordination Facility (CCF) at Human Connectome Project organization. This data is not yet available at CCF on https://humanconnectome.org because CCF is still processing it. The Lifespan Human Connectome Project Aging (HCP-A) data is openly available and can be accessed at the Lifespan dataset in NDA: https://nda.nih.gov/general-query.html?q=query=featured-datasets:HCP%20Aging%20and%20Development. Commercial application software used for the analysis includes FSL, SPM, CONN, FreeSurfer and MATLAB. Custom scripts for data management are available upon request.

## Ethics statement

The studies involving human participants were reviewed and approved by the Institutional Review Board at University of Michigan and the Institutional Review Board at University of Southern California. The patients/participants provided their written informed consent to participate in this study.

## Author contributions

NN, NS, JC, VP, YS, JJ, and JW contributed to the experimental design. NN, NS, JC, VP, HA, YS, and JW performed the data collection. NN, ZL, and JW carried out the data analysis. NS, HA, ZL, JJ, and JW contributed to manuscript editing. NN drafted the manuscript. All authors contributed to the article and approved the submitted version.
