# Supplementary material for: Sight restoration reverses blindness-induced cross-modal functional connectivity changes between the visual and somatosensory cortex at rest
Source: Front Neurosci. 2022 Sep 23;16:902866. doi: 10.3389/fnins.2022.902866 (PMC9539921; doi:10.3389/fnins.2022.902866)
Supplement: Supplementary file 1 [file Data_Sheet_1.docx]

Supplementary Material

# Demographic Information

The age and gender information for the participants along with their respective groups of study are presented below in Supplementary Table 1.

**Supplementary Table 1 - Test Subjects Demographic Information.** Abbreviations: HC = healthy control, RP = retinitis pigmentosa, USC = University of Southern California, UM = University of Michigan and HCP-A = Human Connectome Project, Aging.

| Subject | Group | Data Source | Age | Gender |
| --- | --- | --- | --- | --- |
| 1 | HC | USC | 36 | F |
| 2 | HC | USC | 44 | M |
| 3 | HC | USC | 64 | F |
| 4 | HC | HCP-A | 64 | M |
| 5 | HC | HCP-A | 38 | M |
| 6 | HC | HCP-A | 45 | F |
| 7 | HC | HCP-A | 53 | M |
| 8 | HC | HCP-A | 54 | F |
| 9 | HC | HCP-A | 75 | F |
| 10 | HC | HCP-A | 72 | M |
| 11 | RP | USC | 57 | M |
| 12 | RP | USC | 52 | M |
| 13 | RP | USC | 55 | M |
| 14 | RP | USC | 44 | F |
| 15 | RP | USC | 24 | M |
| 16 | RP | USC | 48 | M |
| 17 | RP | USC | 40 | M |
| 18 | RP | USC | 70 | F |
| 19 | RP | USC | 60 | F |
| 20 | RP | USC | 61 | M |
| 21 | Argus II | USC | 62 | F |
| 22 | Argus II | USC | 46 | M |
| 23 | Argus II | USC | 61 | F |
| 24 | Argus II | USC | 65 | M |
| 25 | Argus II | USC | 76 | M |
| 26 | Argus II | UM | 70 | M |
| 27 | Argus II | UM | 71 | F |

Note: The data was acquired from 1 subject before and after the retinal prosthesis implantation surgery. This is included as subject 19 (before) and 23 (after) in Table 1.

# Clinical Information

| Subject | Group | Duration of Blindness (years)  **Supplementary Table 2 – RP and Argus II Test Subjects Clinical Information.** Abbreviations: LP = light perception, NLP = no light perception, R = right, L = left, N/A = not available. |  |  |  |  |  |  |  |
| --- | --- | --- | --- | --- | --- | --- | --- | --- | --- |
| 11 | RP | 24 |  |  |  |  |  |  |  |
| 12 | RP | 24 |  |  |  |  |  |  |  |
| 13 | RP | 51 |  |  |  |  |  |  |  |
| 14 | RP | 37 |  |  |  |  |  |  |  |
| 15 | RP | 17 |  |  |  |  |  |  |  |
| 16 | RP | N/A |  |  |  |  |  |  |  |
| 17 | RP | 35 |  |  |  |  |  |  |  |
| 18 | RP | 52 |  |  |  |  |  |  |  |
| 19 | RP | N/A |  |  |  |  |  |  |  |
| 20 | RP | 30 |  |  |  |  |  |  |  |
| Subject | Group | Duration of Blindness (years) | Duration of Prosthetic Use (months) | Frequency of Device Usage (days/week) | Total Rehabilitation Training (hours) | Did Subject attend Univ or Second Sight for additional testing? | Vision Left | Vision Right | Eye with Implant |
| 21 | Argus II | 17 | 55 | 7 | 12 | Yes, visited Second Sight several times for 1 week visits | LP | NLP | R |
| 22 | Argus II | 20 | 19.5 | 3.5 | 24 | Yes, visited Second Sight once | LP | LP | R |
| 23 | Argus II | N/A | 6.5 | N/A | N/A | Yes, visited USC | LP or less | LP or less | L |
| 24 | Argus II | 26 | 23 | 1 | 30 | Yes, visited Second Sight five times for 1 week visits | LP | NLP | R |
| 25 | Argus II | 38 | 19 | 7 | 13 | Yes, visited University of Minnesota four times for 1-2 days each | NLP | NLP | R |
| 26 | Argus II | 38 | 48 | 7 | -6 | Yes, visited second sight at least once for a 1 week visit | LP | LP | L |
| 27 | Argus II | 19 | 69 | 2 | -3 | N/A | NLP | NLP | L |

# Summary of the Results of the MRI Acquisition Harmonization between UM and USC Centers

One challenge in merging multicenter neuroimaging data is to derive standardized data features that best signify each center’s performance in spite of the inevitable variation in acquisition due to scanners or adopted procedures. In the current work, we aimed to harmonize the acquisition of the magnetic resonance imaging (MRI) data involved in Human Connectome Low Vision (HCLV) project at two imaging centers: University of Southern California (USC) and University of Michigan (UM). To achieve this goal, we executed a case study in which we recruited a traveling subject, a normally-sighted male adult, at both locations. One T1-weighted (T1W) structural and six block-design task functional MRI runs were acquired with identical paradigms at each center. At USC, a 3T Siemens Prisma scanner was used with voxel size 2x2x2 mm (functional) and 0.8x0.8x0.8 mm (structural) and a multiband (MB) factor of 8 for functional runs. At UM, a 3T MR750 GE scanner was used with MB factor of 6, voxel size 2.4x2.4x2.4 mm (functional) and 0.5x0.5x0.8 mm (structural). Preprocessing consisted of homogeneity, physiological noise, field map, motion and slice-timing corrections as well as smoothing, high-pass filtering, registration to T1 MNI152 template. One T1W and one fMRI run were also obtained from a spherical water-based fBIRN phantom filled with agar gel at each center. In order to quantify the performance of the different scanners, we calculated spatial SNR (Signal-to-Noise-Ratio), spatial CNR (Contrast-to-Noise-Ratio; for gray matter (GM) vs white matter (WM) vs CSF) and temporal SNR. In order to make an equitable assessment between the two centers, same repetition time (TR = 0.8 s) and number of volumes were used for each pair of functional runs; also, the spatial and temporal SNR/CNR values were normalized per unit volume to account for differences in voxel sizes at two centers (nSNR/nCNR). Mean spatial nSNR was 42.9/317.1 (human/phantom) at UM and 25.8/54.6 (human/phantom) at USC. Mean spatial nCNR values for GM-WM, GM-CSF, WM-CSF contrasts were respectively 23.6, 15.6 and 39.3 at UM and 9.7, 11.7 and 21.4 at USC. Mean temporal nSNR across voxels of the subject at both locations yielded comparable results (mean of 3.5 vs 3.7 (UM vs USC) across all tasks), whereas this quantity for phantom data was 8.3 vs 16.8 (UM vs USC).

# Subject Outlier Identification

Inspection of the ROI-ROI rsFC value between V1 ROI and the rest of the brain for Argus II subjects appeared to reveal a visually noticeable difference in connectivity values for one of Argus II subjects (subject A1 in Supplementary Figure 1) compared to the rest of the Argus II subjects. In order to further quantify this apparent difference and investigate the possibility of an outlier subject, we conducted the following analysis.


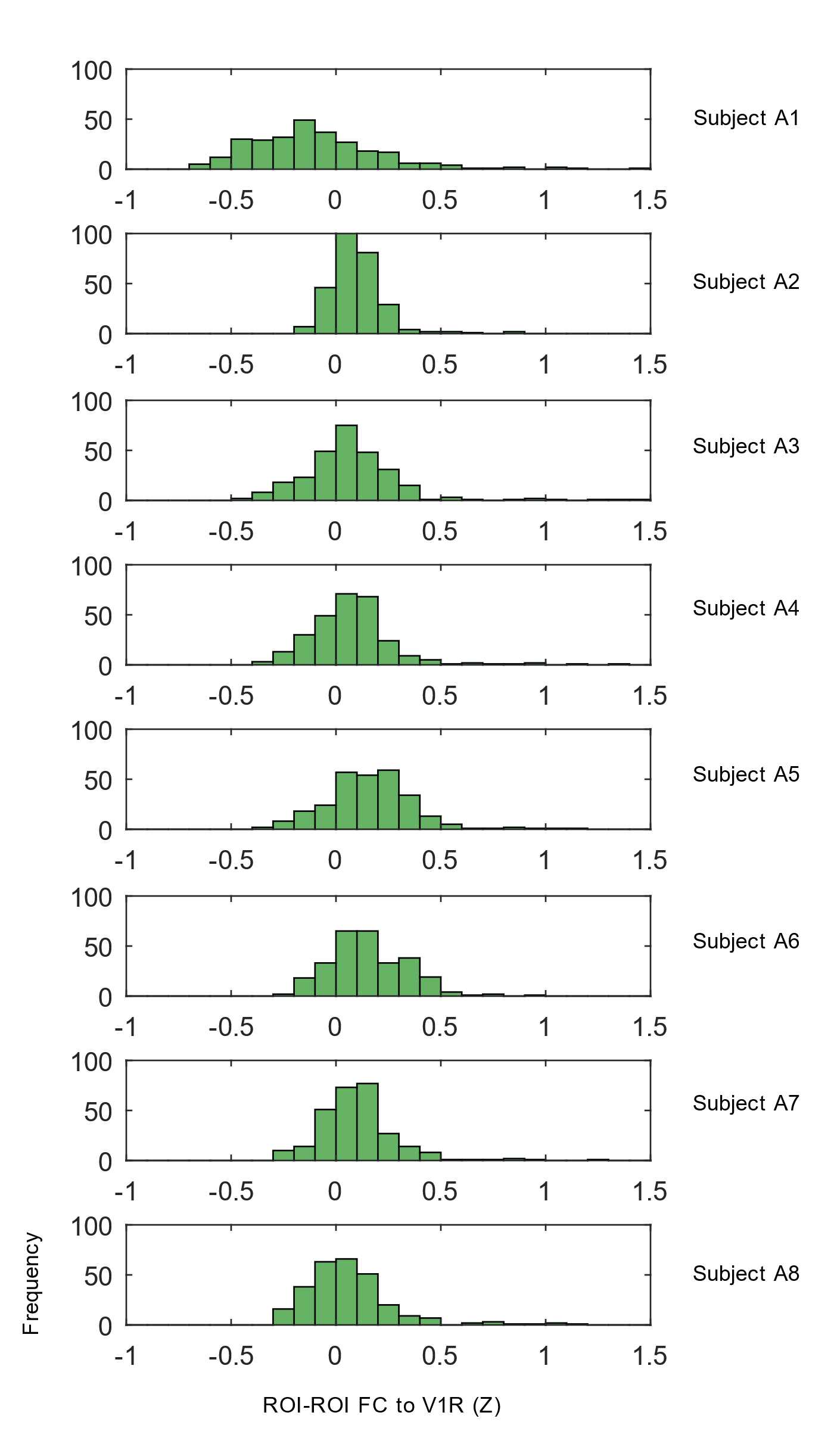


**Supplementary Figure 1 - Distribution of FC Values between V1R and the Rest of the ROIs.** Each panel shows the histogram representing the distribution of ROI-ROI FC values (Z) between V1R ROI and the rest of the ROIs (a total of 282 ROIs as defined by 2020 Julich-Brain atlas (V2.9)) for each of Argus II subject.

The two sample Kolmogorov-Smirnov (Massey, 1951) (K-S) test was applied to the FC values for each pair of the 8 Argus II subjects. The result of the test along with the corresponding significance values are presented in Supplementary Table 2. Although the K-S test resulted in significant differences for many subject pairs, the p-values involving subject 1 (the first 7 rows in Supplementary Table 2) appears to be much smaller than the p-values of other pairs. In order to further look into this difference, we computed the Kernel Density Estimate (KDE) of the 28 p-values shown in Supplementary Table 2 to non-parametrically estimate the probability density function of these p-values. The resulting KDE is presented in Supplementary Figure 2.

**Supplementary Table 3 – K-S Tests Applied to FC values between Subject Pairs (seed: V1R).** Each row shows the result of applying a 2-sample K-S test to each pair of subjects’ FC values, chosen from 8 Argus II subjects. A significant result shows a significant difference between the FC values of the two tested subjects.

| Subject X | Subject Y | Result of K-S test  (1=significant, 0=not significant) | p-value |
| --- | --- | --- | --- |
| 1 | 2 | 1 | 6.97089309993593e-38 |
| 1 | 3 | 1 | 6.93239843710648e-20 |
| 1 | 4 | 1 | 1.35924974213780e-20 |
| 1 | 5 | 1 | 3.54802990051738e-33 |
| 1 | 6 | 1 | 5.07240627626964e-35 |
| 1 | 7 | 1 | 2.20911730868039e-31 |
| 1 | 8 | 1 | 3.58561896807544e-18 |
| 2 | 3 | 1 | 0.000225142319645637 |
| 2 | 4 | 1 | 0.000459224709912611 |
| 2 | 5 | 1 | 4.79676489906896e-11 |
| 2 | 6 | 1 | 1.07714885509768e-06 |
| 2 | 7 | 0 | 0.171026625615983 |
| 2 | 8 | 1 | 4.24562726253443e-07 |
| 3 | 4 | 0 | 0.807870160297195 |
| 3 | 5 | 1 | 6.04460383474617e-08 |
| 3 | 6 | 1 | 2.65444757082276e-06 |
| 3 | 7 | 1 | 0.0232395981009300 |
| 3 | 8 | 0 | 0.342271446958233 |
| 4 | 5 | 1 | 1.55841627759349e-10 |
| 4 | 6 | 1 | 1.07714885509767e-06 |
| 4 | 7 | 1 | 0.0298681593025658 |
| 4 | 8 | 0 | 0.245735550358943 |
| 5 | 6 | 0 | 0.291177067509870 |
| 5 | 7 | 1 | 4.24562726253445e-07 |
| 5 | 8 | 1 | 2.21988668722729e-12 |
| 6 | 7 | 1 | 0.000909825128536422 |
| 6 | 8 | 1 | 1.50746508283792e-09 |
| 7 | 8 | 1 | 0.000322716091874584 |


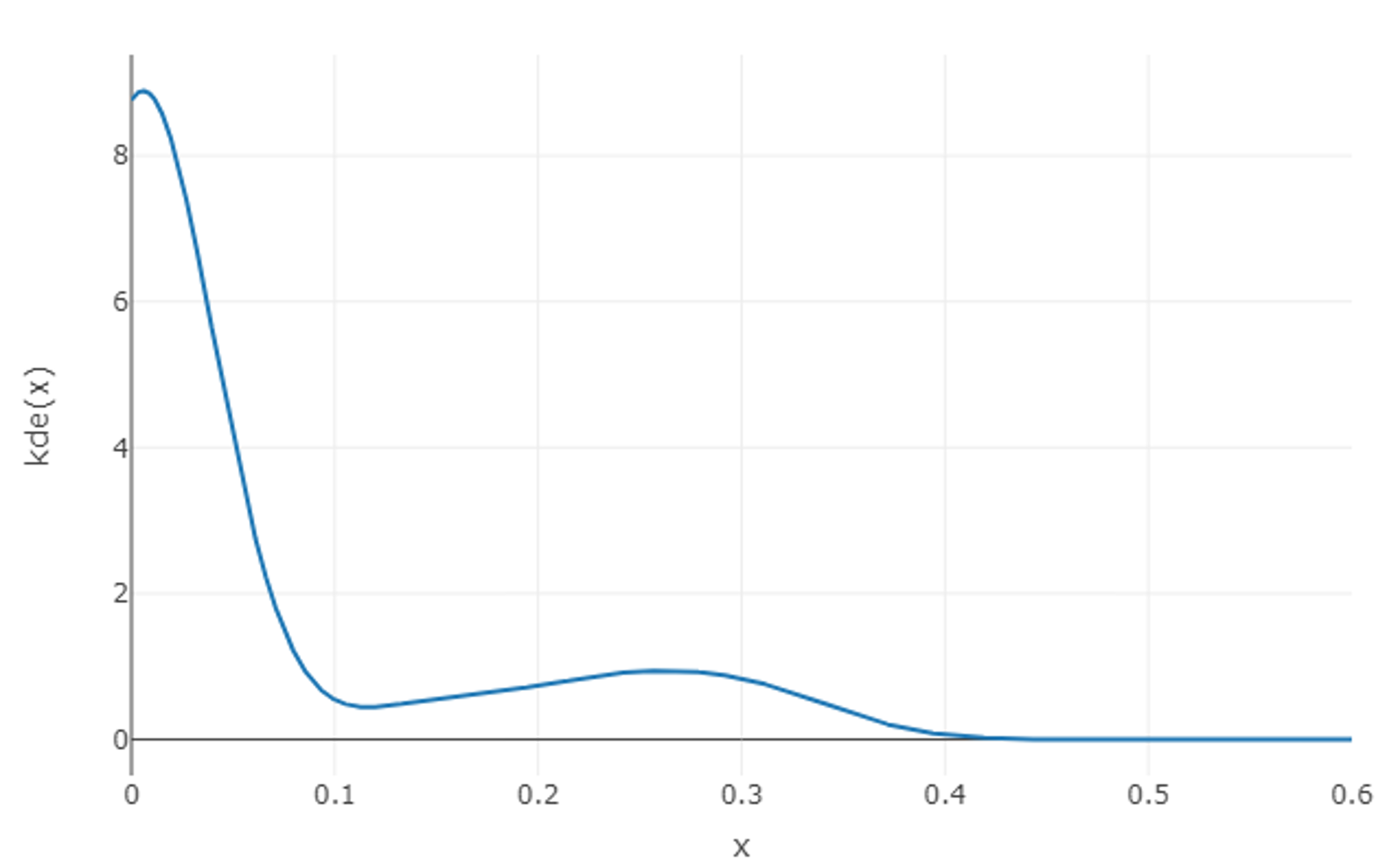


**Supplementary Figure 2 – Kernel Density Estimate of the p-values (seed: V1R).** The KDE was computed for all the p-values presented in Supplementary Table 2. X-axis represents the p-values.

As indicated in Supplementary Equation 1, we integrated the KDE function presented in Supplementary Figure 2, for the p-values involving subject 1. The result was an incredibly small probability value of 3.14 x 10^-17^, indicating that **subject 1** is very far from all other participants in this study. This is strong evidence that subject 1 may be an outlier.

| $P_{1:8} \left( x<p\left[ 7 \right] \right)= {CDF}_{1:8}\left( 3.58 x {10}^{-18} \right)= \int_{0}^{3.58 x {10}^{-18}} kde\left( x \right)dx=3.14 x {10}^{-17}$ | **(Supplementary Equation 1)** |
| --- | --- |

We repeated the same analysis for V1L (V1 left) ROI as the seed as indicated in Supplementary Figures 3, Table 3 and Figure 4. The investigation resulted in similar outcome as indicated in Supplementary Equation 2. Consequently, Argus II subject 1 was removed from the rest of the analysis for rsFC.


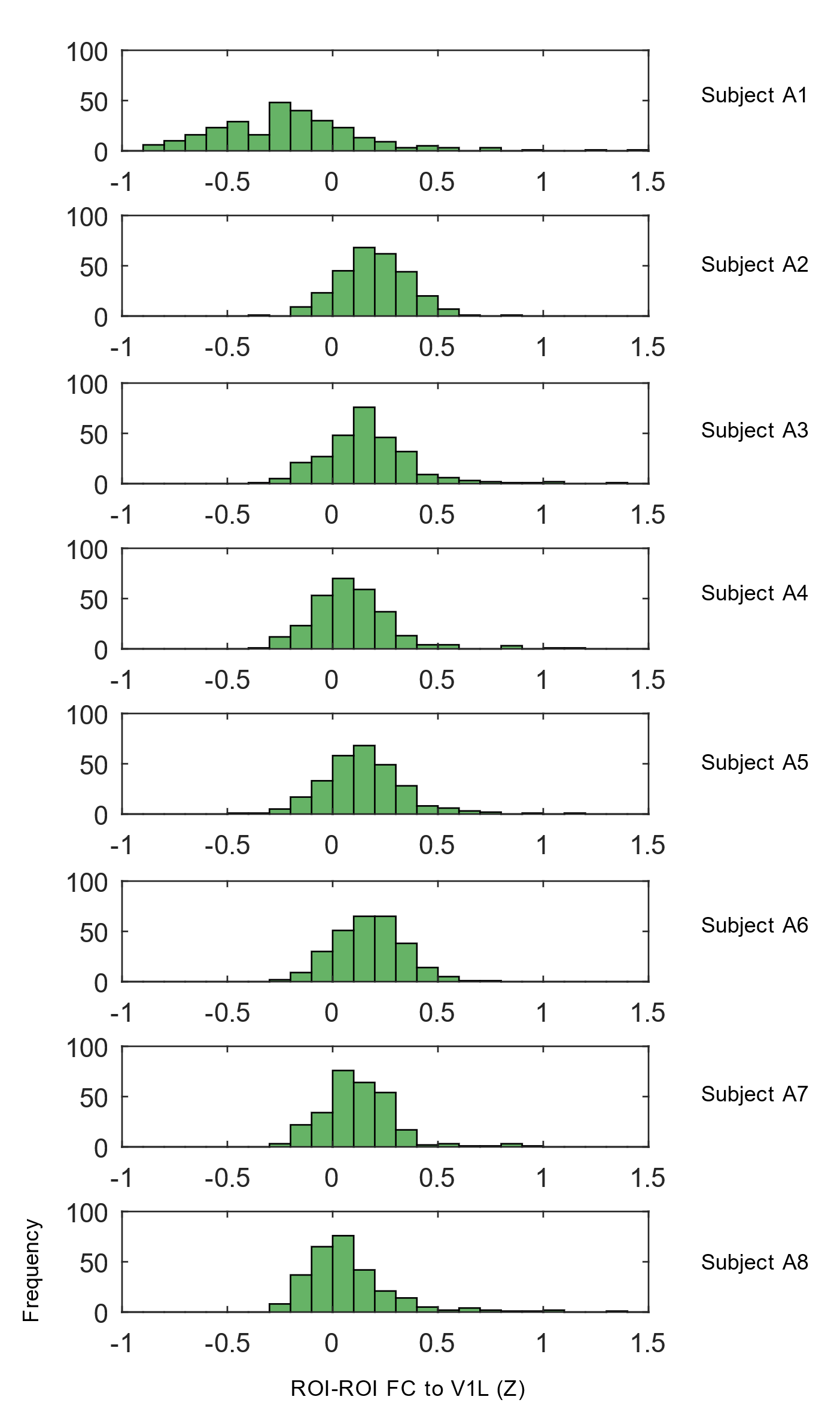


**Supplementary Figure 3 - Distribution of FC Values between V1L and the Rest of the ROIs.** Each panel shows the histogram representing the distribution of ROI-ROI FC values (Z) between V1L ROI and the rest of the ROIs (a total of 282 ROIs as defined by 2020 Julich-Brain atlas (V2.9)) for each of Argus II subject.

**Supplementary Table 4 -** **K-S Tests Applied to FC values between Subject Pairs (seed: V1L).** Each row shows the result of applying a 2-sample K-S test to each pair of subjects’ FC values, chosen from 8 Argus II subjects. A significant result shows a significant difference between the FC values of the two tested subjects.

| Subject A | Subject B | Result of K-S test  (1=significant, 0=not significant) | p-value |
| --- | --- | --- | --- |
| 1 | 2 | 1 | 2.94356628930396e-59 |
| 1 | 3 | 1 | 3.03552098721553e-48 |
| 1 | 4 | 1 | 6.97089309993593e-38 |
| 1 | 5 | 1 | 1.07224777262513e-47 |
| 1 | 6 | 1 | 1.92148901841483e-57 |
| 1 | 7 | 1 | 1.30900831014120e-46 |
| 1 | 8 | 1 | 5.07240627626964e-35 |
| 2 | 3 | 1 | 0.0381090965854382 |
| 2 | 4 | 1 | 7.75629702972784e-12 |
| 2 | 5 | 1 | 0.00175087512674723 |
| 2 | 6 | 0 | 0.171026625615983 |
| 2 | 7 | 1 | 4.24562726253445e-07 |
| 2 | 8 | 1 | 3.43425341613383e-19 |
| 3 | 4 | 1 | 4.12178256834523e-06 |
| 3 | 5 | 0 | 0.460999028158125 |
| 3 | 6 | 0 | 0.205784138661939 |
| 3 | 7 | 1 | 0.0104793476841627 |
| 3 | 8 | 1 | 2.21988668722730e-12 |
| 4 | 5 | 1 | 7.31835598435305e-05 |
| 4 | 6 | 1 | 1.29798392677044e-08 |
| 4 | 7 | 1 | 0.00175087512674723 |
| 4 | 8 | 0 | 0.115524418832263 |
| 5 | 6 | 0 | 0.0606996862648646 |
| 5 | 7 | 0 | 0.0939042216839960 |
| 5 | 8 | 1 | 7.66041130793069e-09 |
| 6 | 7 | 1 | 1.47728642708130e-05 |
| 6 | 8 | 1 | 1.11347885308774e-14 |
| 7 | 8 | 1 | 1.69708801772414e-06 |


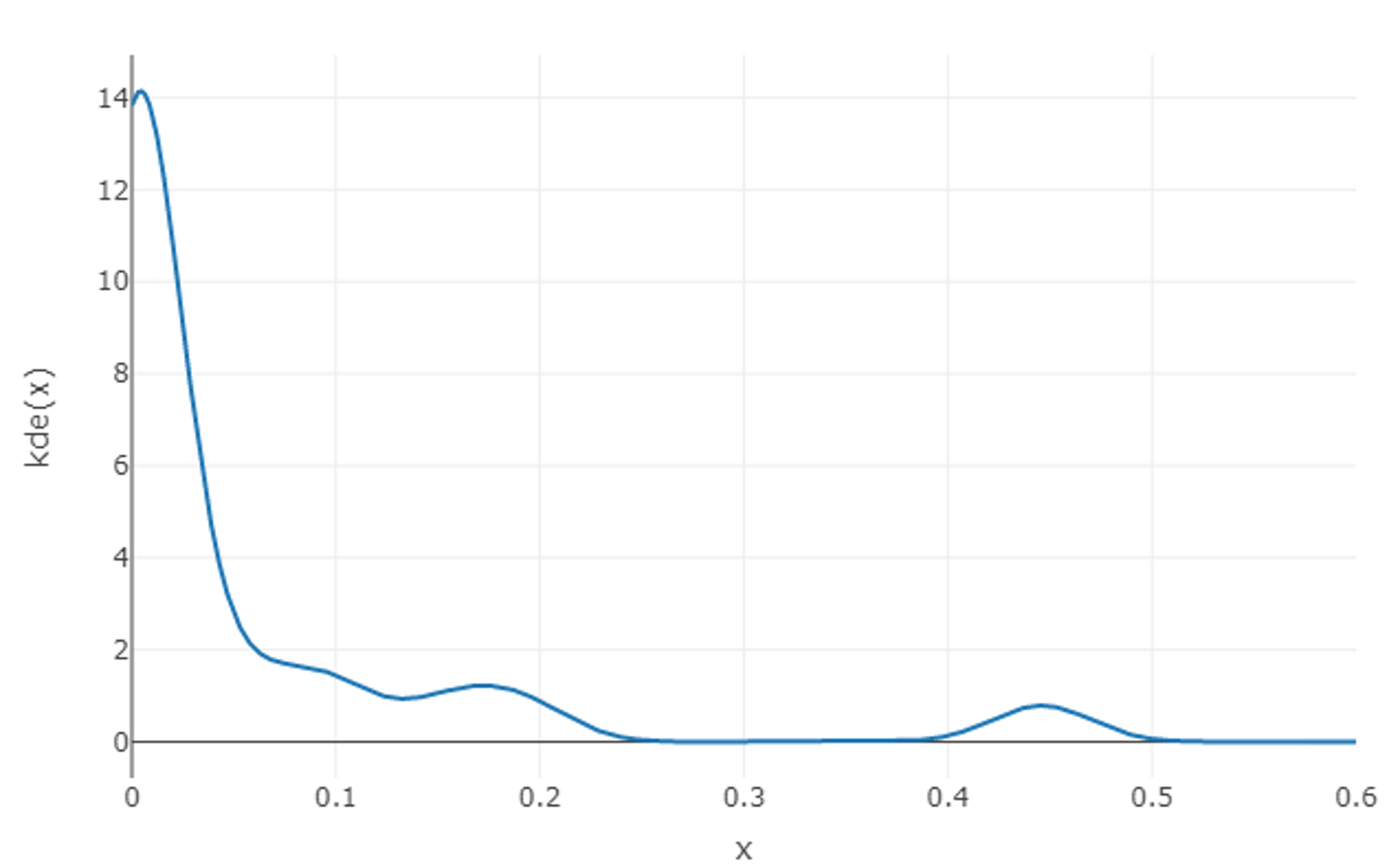


**Supplementary Figure 4 -** **Kernel Density Estimate of the p-values (seed: V1L).** The KDE was computed for all the p-values presented in Supplementary Table 3. X-axis represents the p-values.

| $P_{1:8} \left( x<p\left[ 7 \right] \right)= {CDF}_{1:8}\left( 5.07 x {10}^{-35} \right)= \int_{0}^{5.07 x {10}^{-35}} kde\left( x \right)dx=7.012714 x {10}^{-34}$ | **(Supplementary Equation 2)** |
| --- | --- |

# Normality Test, Effect Size and Power

5.A) Normality Test

For each of the 3 subject groups (or a combination of the groups), for each voxel, a two-sample Kolmogorov-Smirnov (K-S) test (Massey, 1951) was applied for comparison with a normal distribution with the same mean and standard deviation (s.d.), as follows.

K-S test for each voxel (between HC + RP + Argus II and a normal distribution with the same mean and s.d.):

Number of voxels with significant results / percentage (uncorrected): 198 / 8.3961e-04%

Number of voxels with significant results / percentage (corrected): 0 / 0

K-S test for each voxel (between HC and a normal distribution with the same mean and s.d.):

Number of voxels with significant results / percentage (uncorrected): 14 / 5.9366e-05%

Number of voxels with significant results / percentage (corrected): 0 / 0

K-S test for each voxel (between RP and a normal distribution with the same mean and s.d.):

Number of voxels with significant results / percentage (uncorrected): 32 / 1.3569e-04%

Number of voxels with significant results / percentage (corrected): 0 / 0

K-S test for each voxel (between Argus II and a normal distribution with the same mean and s.d.):

Number of voxels with significant results / percentage (uncorrected): 1 / 4.2404e-06%

Number of voxels with significant results / percentage (corrected): 0 / 0

5. B) Effect Size

The ROI-to-ROI analysis for RP > HC contrast resulted is statistically significant result with an effect size of -0.45. Another previous studies that looked at the effect of blindness in late blind vs healthy control showed an approximate effect size of -0.15 between left/right V1 and Pre/Post central gyrus (Wen et al., 2018). The ROI-to-ROI analysis for Argus II > RP contrasts resulted is statistically significant result with an effect size of 0.28. To our knowledge, no sight restoration study has quantified such effect size prior to this work. The ROI-to-ROI analysis for Argus II > HC did not result in any significant result.

5. C) Power Analysis:

For power analysis for RP > HC contrast, we used an approximate effect size of -0.15 between the left/right V1 and Pre/Post central gyrus, obtained from an older study on the effect of late blindness (Wen et al., 2018). We also used the sample size of our study (10 (HC) + 10 (RP) – 2) and the desired type I error rate (0.05). Therefore, assuming an effect size of -0.15, a statistical power of 0.95 was yielded for this contrast. For Argus II > RP contrast, to our knowledge, no sight restoration study has quantified such effect size prior to this work.

**References**

Massey, F. J. (1951). The Kolmogorov-Smirnov Test for Goodness of Fit. *Journal of the American Statistical Association*, *46*(253), 68–78.

Wen, Z., Zhou, F.-Q., Huang, X., Dan, D., Xie, B.-J., & Shen, Y. (2018). Altered functional connectivity of primary visual cortex in late blindness. *Neuropsychiatric Disease and Treatment*. https://doi.org/10.2147/NDT.S183751
